# Molecular profiling of pre- and post-treatment pediatric high-grade astrocytomas reveals acquired increased tumor mutation burden in a subset of recurrences

**DOI:** 10.1186/s40478-023-01644-4

**Published:** 2023-09-05

**Authors:** Matthew D. Wood, Carol Beadling, Tanaya Neff, Steve Moore, Christina A. Harrington, Lissa Baird, Christopher Corless

**Affiliations:** 1 Department of Pathology and Laboratory Medicine, Oregon Health & Science University, 3181 SW Sam Jackson Park Road, L-113, Portland, OR 97239 USA; 2 Knight Cancer Institute, Oregon Health & Science University, Portland, OR USA; 3 Department of Molecular and Medical Genetics, Oregon Health & Science University, Portland, OR USA; 4 Integrated Genomics Laboratory, Oregon Health & Science University, Portland, OR USA; 5 Department of Neurological Surgery, Oregon Health & Science University, Portland, OR USA; 6 Present Address: Boston Children’s Hospital, Boston, MA USA

**Keywords:** High-grade astrocytoma, Pediatric brain tumor, Hypermutation, DNA mismatch repair, Molecular profiling

## Abstract

Diffuse gliomas are a heterogeneous category of primary central nervous system tumors. Due to their infiltrative growth precluding complete surgical resection, most diffuse high-grade gliomas are treated with adjuvant chemotherapy and radiation. Recurrent/progressive diffuse gliomas may show genetic differences when compared to the primary tumors, giving insight into their molecular evolution and mechanisms of treatment resistance. In adult-type diffuse gliomas with or without isocitrate dehydrogenase gene mutations, tumor recurrence/progression can be associated with mutations in genes encoding DNA mismatch repair proteins, leading to a dramatic increase in tumor mutation burden. This phenomenon is closely linked to treatment with the DNA alkylating agent temozolomide, a mainstay of adult diffuse glioma chemotherapeutic management. Post-treatment mismatch repair deficiency and acquired high tumor mutation burden is relatively unexplored in pediatric patients who have recurrent high-grade gliomas. Here, we report a molecular and histological analysis of an institutional cohort of eleven pediatric patients with paired initial and recurrent high-grade astrocytoma samples with intervening temozolomide treatment. We identified three cases with evidence for increased tumor mutation burden at recurrence, including two cases of diffuse hemispheric glioma H3 G34-mutant (one previously reported). We also show that molecular analysis by next-generation DNA sequencing and DNA methylation-based profiling enabled an integrated diagnosis per 2021 World Health Organization criteria in 10 of 11 cases (91%). Our findings indicate that increased tumor mutation burden at post-treatment recurrence is relevant in pediatric-type diffuse high-grade gliomas. Diffuse hemispheric glioma H3 G34-mutant may be particularly susceptible to this phenomenon.

**Supplementary Information:**

The online version contains supplementary material available at 10.1186/s40478-023-01644-4.

## Introduction

Central nervous system (CNS) tumors are the most common solid tumor in children and adolescents [1]. Clinical outcome depends on several factors including tumor location, amenability to surgical resection, and the tumor type as defined by histologic and molecular features. About 10% of pediatric CNS tumors are categorized pathologically as pediatric-type diffuse high-grade gliomas (pHGG) which reflects a growth pattern of diffusely infiltrative tumor cells accompanied by aggressive histologic features including cytologic anaplasia, increased mitotic activity, microvascular proliferation, and necrosis. This category includes diffuse midline glioma H3 K27-altered (DMG), diffuse hemispheric glioma H3 G34-mutant (DHG), and diffuse pediatric-type high-grade glioma H3-wildtype and isocitrate dehydrogenase (IDH)-wildtype [2]. Other high-grade astrocytic gliomas that can present in children and adolescents are pleomorphic xanthoastrocytoma World Health Organization (WHO) grade 3 and high-grade astrocytoma with piloid features—both of which have circumscribed growth—and the infant-type hemispheric glioma group which is largely defined by demographics, epigenetic profiling, and receptor tyrosine kinase gene rearrangements [3].

The molecular features of pediatric-type diffuse gliomas differ from the adult-type diffuse gliomas. Most adult-type diffuse gliomas are accounted for by IDH-mutant astrocytoma (CNS WHO grade 2, 3, or 4), IDH-mutant and 1p/19q-codeleted oligodendroglioma (CNS WHO grade 2 or 3), and IDH-wildtype glioblastoma (CNS WHO grade 4). The standard treatment for adult-type diffuse gliomas depends on tumor type and grade [4]. For higher-grade and/or incompletely resected tumors, treatment includes maximum safe surgical resection followed by combined radiation therapy with adjuvant chemotherapy and maintenance chemotherapy. The DNA alkylating agent temozolomide (TMZ) is used in the treatment of adult-type diffuse gliomas and is most commonly applied in the settings of higher-grade (i.e. 3 or 4) IDH-mutant astrocytoma and in IDH-wildtype glioblastoma. Optimal treatment of pediatric-type diffuse gliomas is not fully established and a role for TMZ in management of pediatric-type diffuse gliomas is debated [5].

Due to their infiltrative growth pattern, diffuse gliomas cannot be completely resected and eventually recur in most cases. Diffuse glioma recurrence can be associated with an increase in the tumor’s histologic grade (sometimes denoted as “progression”), correlating with newly-acquired genetic changes that impact cell proliferation, cell survival, and cell cycle regulation [6]. A subset of adult-type diffuse gliomas that have been exposed to TMZ can show a markedly increased tumor mutation burden (TMB) at recurrence/progression, with a distinct signature of DNA mutations that result from DNA replication across unrepaired TMZ-DNA adducts [7]. This mutational signature is associated with defects in the DNA mismatch repair (MMR) proteins, which may be associated with somatic MMR gene mutations identified in recurrent tumors that are not detected in the initial resection [8].

High tumor mutation burden in pediatric cancer patients is most commonly studied in the setting of germline DNA replication/repair defects [9, 10]. The phenomenon of acquired, post-treatment MMR deficiency in pediatric CNS tumors is comparatively rare. In this study, we examined a single-institution retrospective cohort of 11 pediatric high-grade astrocytic gliomas with matched pre- and post-treatment tumor specimens, including one previously published case from our institution of diffuse hemispheric glioma H3 G34-mutant that was found to have treatment-associated MMR deficiency and increased tumor mutation burden [11].

## Materials and methods

Neurosurgery and neuropathology databases at Oregon Health & Science University (OHSU) were searched to identify 72 patients ≤ 18 years old who had a tissue diagnosis of a high-grade primary central nervous system tumor between 2020 and 2022. Patients who had at least two tissue samples diagnostic for tumor at OHSU were identified, and the medical records were reviewed to identify any history of temozolomide exposure in between tumor samplings. Other clinical data were extracted from the electronic medical record. Treatment timing, time to progression, and overall survival were calculated from the date of surgery diagnostic for high-grade features as Day 0.

Pathology slides were reviewed by a neuropathologist (MDW) to confirm the histological diagnosis and assess tissue adequacy for molecular studies. Nucleic acids were extracted from macrodissected tumor-rich areas off unstained sections of formalin-fixed paraffin embedded tissue. When available, archival nucleic acid samples from clinical testing were studied; otherwise, fresh unstained sections were prepared for extractions. Next-generation sequencing (NGS) was performed using a clinically validated next-generation DNA sequencing panel (GeneTrails^®^ solid tumor panel) run on an Illumina NextSeq500/550. Custom sequencing libraries were prepared using a PCR-based technology (QiaSeq, Qiagen, Inc.) that covers 225 genes (200 whole-exon, 25 hotspot, full gene list available at https://knightdxlabs.ohsu.edu/home/test-details?id=GeneTrails+Comprehensive+Solid+Tumor+Panel). The GeneTrails^®^ sequencing footprint is approximately 0.61 Mb and detects single nucleotide variants, small insertions/deletions, copy number alterations, and microsatellite instability status from 226 short tandem repeats. Sequence variants are identified using FreeBayes and MuTect2 algorithms in a custom analysis pipeline.

Immunohistochemical stains for mismatch repair proteins were performed at the OHSU Histopathology Shared Resource. Stains were performed on a Ventana Benchmark XT with the following reagents and conditions: MSH2 Roche G219-1129 (predilute, 12 min incubation), MSH6 Roche SP93 (predilute, 12 min incubation), MLH1 Leica Novocastra MLH1-L-CE (1:25, 24 min incubation), PMS2 BD Pharmagen A16-4 (1:75, 32 min incubation). Antigen retrievals conditions were 40 min Cell Conditioner 1 (CC1, pH 6) for MSH2, 64 min CC1 for MSH6 and MLH1, and 92 min CC1 for PMS2. Antigen detection was performed with the Optiview DAB chromogen detection kit and counter-staining was 4 min incubation in hematoxylin II and 4 min bluing. Stains were interpreted by a neuropathologist (MDW). Loss of MMR protein staining was defined as > 90% of tumor cell nuclei being negative for a stain, with the presence of adequate internal positive control non-neoplastic cell labeling in the same areas.

Genome-wide DNA methylation-based profiling was performed using nucleic acids extracted as described above, followed by a bisulfite conversion protocol. Genome-wide DNA methylation-based classification was done using the online DKFZ methylation classifier version (www.molecularneuropathology.org) [12]. This study was conducted under OHSU Institutional Review Board approval with a waiver of patient consent (STUDY23667), with clinical data obtained under a separate IRB-approved neurosurgery/neuropathology data repository (STUDY18995).

## Results

The cohort is summarized in Table 1 and included 8 male and 3 female patients with median age of 12 years at initial diagnosis (range 6–17 years). Eight tumors were located in the cerebral hemispheres, two were in midline locations (1 pineal region, 1 cingulate gyrus), and one was located in the posterior fossa (cerebellar hemisphere). No patients were known or suspected to have neurofibromatosis type 1. Due to the retrospective nature of this study, most of the pathological diagnoses predated the identification of key molecular drivers of pediatric high-grade gliomas such as H3 K27 and G34 mutations (p.K28/p.G35), and all samples predated the nomenclature and classification updates to pediatric-type diffuse high-grade gliomas from the 2021 5^th^ Edition of the World Health Organization Classification of Central Nervous System Tumors [WHO CNS 5]. Therefore, the diagnoses in the medical record were often descriptive or used legacy nomenclature such as “glioblastoma”, “anaplastic oligoastrocytoma”, or “anaplastic astrocytoma”. Ten of the 11 cases were CNS WHO grade 3 or 4 at the initial surgical intervention. The exception was pTMZ-05, a case of “anaplastic astrocytoma” grade 3 that was a re-resection of a residual/recurrent grade 2 diffuse glioma that had been diagnosed 288 days earlier by biopsy, without intervening chemotherapy or radiation therapy.

**Table 1:** Clinical features, treatment histories, and survival data

Case	Age (years)	Sex	Tumor location	Resection	Tumor type *at diagnosis* or per WHO CNS 5	Chemoradiation	Maintenance Temozolomide	TMZ end to recurrence (days)	Overall survival and (survival from recurrence)
pTMZ-01	14	M	Left temporal	Biopsy	*Infiltrating anaplastic astrocytoma with areas of PNET-like dedifferentiation*	5940 cGy TMZ 90 mg/m^2^ daily for 42 days	10 cycles 150–200 mg/m^2^ daily for 5 days	114	814 (245)
pTMZ-02	16	M	Left parietal	Gross total	*Anaplastic oligoastrocytoma*	5940 cGy TMZ 90 mg/m^2^ daily for 42 days	15 cycles 200 mg/m^2^ daily for 5 days	2306	3584 (740)
pTMZ-03	12	M	Right frontotemporal	Gross total	Diffuse pediatric-type high-grade glioma, H3/IDH-wildtype	6000 cGy TMZ 90 mg/m^2^ daily for 42 days	17 cycles 150 mg/m^2^ daily for 5 days	0 [progressed on maintenance therapy]	1044 (406)
pTMZ-04	12	F	Right frontal	Gross total	Diffuse pediatric-type high-grade glioma, H3/IDH-wildtype	6000 cGy TMZ 160 mg daily for 42 days	18 cycles 200 mg/m^2^ daily for 5 days	905	1839 (293)
pTMZ-05	6	M	Right cingulate	Gross total	*Anaplastic astrocytoma*	5940 cGy TMZ 90 mg/m^2^ daily for 42 days	18 cycles 90 mg/m^2^ daily for 5 days	744	1639 (255)
pTMZ-06	11	M	Right frontotemporal	Biopsy	*Anaplastic astrocytoma*	Radiation details not available TMZ 100 mg daily for 42 days	1300 mg/cycle Number of cycles not available	397	892 (132)
pTMZ-07	16	F	Left frontal	Gross total	Diffuse hemispheric glioma, H3 G34-mutant	6000 cGy TMZ 90 mg/m^2^ daily for 42 days	18 cycles 200 mg/m^2^ daily for 5 days	473	1433 (376)
pTMZ-08	13	M	Pineal region	Gross total	*Atypical mixed glioneuronal neoplasm with aggressive features, arising in a background of papillary glioneuronal tumor*	5940 cGy TMZ 90 mg/m^2^ daily for 21 days	3 cycles 200 mg/m^2^ daily for 5 days	0 [progressed on maintenance therapy]	473 (275)
pTMZ-09	8	M	Right temporal	Subtotal	*Glioblastoma*	Chemoradiation details not available	12 cycles 1300 mg/cycle	183	1086 (419)
pTMZ-10	11	M	Right temporal	Gross total	*Anaplastic astrocytoma*	5940 cGy TMZ 120 mg daily for 28 days	None	315	728 (657)
pTMZ-11	17	F	Right cerebellum	Subtotal	Astrocytoma, IDH-mutant, CNS WHO grade 4	5940 cGy TMZ 160 mg daily for 41 days	Maintenance details not available	894	2094 (628)

**Fig. 1:**
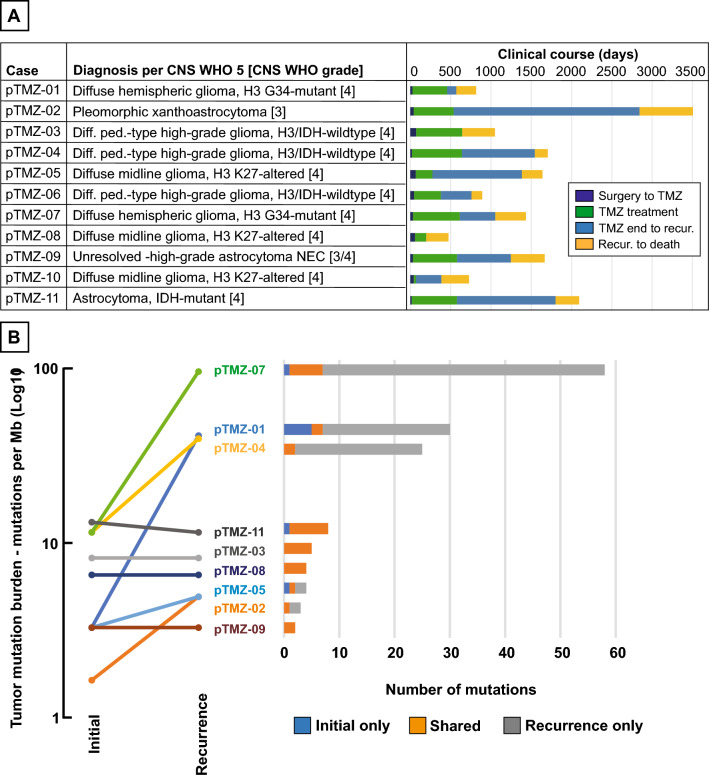
Treatment courses and pre/post-treatment mutation burden in pHGG. **A** Tumor diagnoses based on histological, genetic, and epigenetic profiling of samples in this study with treatment summaries. **B** Pre- and post-treatment tumor mutation burden for 9 cases with matched pair next-generation sequencing results

Treatment regimens included maximum safe surgical resection followed by combined chemoradiotherapy and maintenance TMZ (Table 1). In general, combined chemoradiation therapy with 5940 or 6000 cGy of fractionated photon radiation was delivered concurrently with 90 mg/m^2^ of TMZ for up to 42 days. Chemoradiation treatment details were not available for case pTMZ-09. One patient stopped TMZ during chemoradiotherapy due to severe thrombocytopenia and did not receive maintenance chemotherapy [pTMZ-10]. Ten patients started maintenance TMZ with 8 having evaluable treatment records (documentation incomplete or unavailable for pTMZ-06 and pTMZ-11, respectively). Two patients had clinical or radiographic tumor progression during TMZ maintenance therapy [pTMZ-03 and pTMZ-08]. The 6 patients who completed their courses of maintenance TMZ and had available records received between 10 and 18 treatment cycles and had duration from the end of maintenance TMZ to disease progression ranging from 114 to 2306 days (mean 703 days). All patients in the cohort were deceased, with overall survival from their first high-grade tumor diagnosis ranging from 473 to 3584 days (median 1086 days) and survival from the time of post-treatment recurrence ranging from 132 to 740 days (median 376 days). Clinical information is summarized schematically in Fig. 1A and radiologic features are presented in Additional file 1: Fig. S1.

When incorporating information available in the clinical record from all specimens for each patient, 4 of 11 cases had sufficient data to retrospectively assign an integrated diagnosis per WHO CNS5. This included one astrocytoma IDH-mutant (CNS WHO grade 4) [pTMZ-11], one diffuse hemispheric glioma H3 G34-mutant [pTMZ-07], and two cases of diffuse pediatric-type high-grade glioma H3-wildtype and IDH-wildtype [pTMZ-03 and -04] (Table 1). Genetic findings were used to update the diagnostic categories for the remaining 7 cases. Sequencing and copy number data allowed for a modernized diagnosis in 6 of 7 tumors (Table 2). This included three cases of diffuse midline glioma H3 K27-altered [pTMZ-05, -08, and -10], one diffuse hemispheric glioma H3 G34-mutant [pTMZ-01], one diffuse pediatric-type high-grade glioma H3/IDH-wildtype [pTMZ-06], and one pleomorphic xanthoastrocytoma CNS WHO grade 3 [pTMZ-02]. Integrated diagnoses were supported by the results of genome-wide DNA methylation-based profiling (DNA-MP) for most of the cases (Table 2, and Additional file 2: Table S1). Ten cases had DNA-MP performed on at least one tumor sample, with 7 cases showing a classifier match with calibrated score > 0.9 for initial and/or recurrent tumor based on v12.5 of the DKFZ brain tumor classifier. Six of 10 cases had *MGMT* promoter hypermethylation on methylation array analysis, and 8 cases with paired methylation array data had the consistent *MGMT* methylation status in both the pre- and post-treatment samples (Additional file 2: Table S1). Overall, when incorporating the genetic, epigenetic, and histological findings, 10 of 11 tumors in the cohort (91%) were successfully placed into a modern WHO CNS 5 classification. The unresolved case, pTMZ-09, was found to have a pathogenic *TP53* frameshift mutation, *BRAF* p.V600E mutation, and homozygous deletion of *CDKN2A* in both the pre- and post-TMZ samples. This genetic profile was interpreted as compatible with epithelioid glioblastoma or grade 3 pleomorphic xanthoastrocytoma. Although the histologic features aligned best with glioblastoma and this was the historical diagnosis from the medical record (pictured in Additional file 3: Fig. S2), neither the initial nor the recurrent tumor matched to a specific methylation class. Therefore, pTMZ-09 was designated in this study as a high-grade astrocytoma not elsewhere classified (NEC).

**Table 2:** Modernized neuropathological diagnosis based on molecular results

Case	Original diagnosis	Genetic findings	Integrated diagnosis per WHO CNS5	DNA methylation-based classification
pTMZ-01	Infiltrating anaplastic astrocytoma with areas of PNET-like dedifferentiation	*H3-3A* G34R (p.G35R)	Diffuse hemispheric glioma, H3 G34-mutant, CNS WHO grade 4	Not available
pTMZ-02	Anaplastic oligoastrocytoma	*BRAF* p.V600E *CDKN2A* homozygous deletion	Pleomorphic xanthoastrocytoma, CNS WHO grade 3	PXA
pTMZ-05	Anaplastic astrocytoma	*H3-3A* K27M (p.K28M) *EGFR* amplification	Diffuse midline glioma, H3 K27-altered, CNS WHO grade 4	DMG_EGFR
pTMZ-06	Anaplastic astrocytoma	IDH and H3 wildtype	Diffuse pediatric-type high-grade glioma, H3-wildtype and IDH-wildtype, CNS WHO grade 4	pedHGG_RTK2A
pTMZ-08	Atypical mixed glioneuronal neoplasm with aggressive features, arising in a background of papillary glioneuronal tumor	*H3-3A* K27M (p.K28M)	Diffuse midline glioma, H3 K27-altered, CNS WHO grade 4	Not available
pTMZ-09	Glioblastoma	*TP53* p.D48fs*71 *BRAF* p.V600E *CDKN2A* homozygous deletion	High-grade astrocytoma, NEC (see text)	No match
pTMZ-10	Anaplastic astrocytoma	*H3-3A* K27M (p.K28M) *EGFR* exon 20 in-frame insertion	Diffuse midline glioma, H3 K27-altered, CNS WHO grade 4	DMG_EGFR

We next analyzed the tumor mutation burden (TMB) in nine cases where pre- and post-TMZ tumor samples were evaluable by NGS. In addition to the index case that we have previously reported [pTMZ-07], we found a marked increase in the post-TMZ tumor mutation burden for 2 additional cases [pTMZ-01 and -04] (Fig. 1B). Although two cases did not have adequate tissue for paired genetic analysis [pTMZ-06 and pTMZ-10], immunohistochemical staining showed intact MMR protein expression in both cases at recurrence and there was no evidence of increased TMB in pTMZ-06 which had adequate tissue for sequencing of the recurrence specimen only [4.9 mutations/Mb]. All genetic variants identified in the entire cohort are listed in Additional file 4: Table S2. Details of the two novel post-treatment TMB-High cases are described subsequently; the features of pTMZ-07 are already reported in the literature [11].

**Fig. 2:**
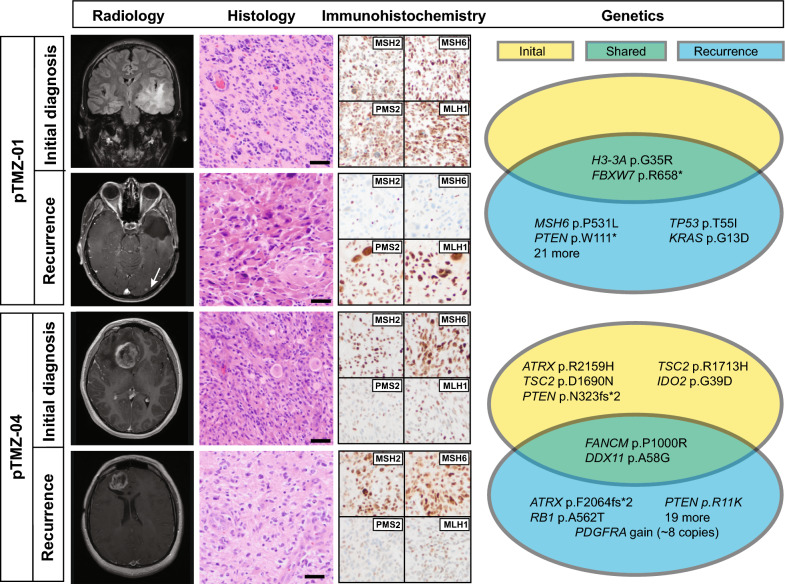
Summary of two newly-identified pHGG cases with increased TMB at recurrence. Radiologic, histologic, immunohistochemical, and genetic features are summarized. Scale bars for histology = 50 microns. Radiology images are T2-weighted FLAIR for pre-treatment pTMZ-01 and T1-weighted post-contrast for all other images

Case pTMZ-01 from a left temporal tumor in a 14-year-old male patient was initially diagnosed by biopsy as an infiltrating astrocytoma with primitive neuroectodermal features and resolved to diffuse hemispheric glioma H3 G34-mutant by sequencing (Fig. 2, top). Subsequent treatment included chemoradiotherapy and 10 cycles of maintenance TMZ. A distant focus of recurrent, disseminated disease was identified in the occipital lobe on surveillance neuroimaging 16 weeks after the last documented TMZ cycle. Despite some histological differences in the occipital lobe tumor, which had striking gemistocytic morphology, both tumors were shown to be *H3-3A* G34R mutant (p.G35R) supporting a common genetic driver. The tumor from the left occipital region was found to have a TMB of 41 mutations/Mb, compared to 3.3 mutations/Mb in the pre-treatment sample. This was associated with an *MSH6* mutation, CCDS1836.1 c.1592C > T, p.P531L, that was undetected in the initial tumor and correlated with loss of MSH6 and MSH2 protein immunoreactivity in the recurrent tumor only (Fig. 2). The p.P531L alteration results in a proline to leucine substitution within a connector domain that links the MSH6 mismatch binding domain to a lever domain, and is within an MSH2 interacting region of MSH6 [13, 14]. Proline 531 resides in a loop within MSH6 that is in proximity to a predicted hydrogen bond between MSH6 and MSH2 (Additional file 5: Fig. S3), and this alteration could be predicted to disrupt the MutSα heterodimer stability. This variant is not reported in population databases (ExAC, gnomAD, ESP, and 1 K Genomes) and has not been reported in cancer databases (ClinVar, COSMIC, cBioPortal) or functionally characterized. The variant allele frequency was 0.56, suggestive of a heterozygous alteration. The possibility of other, undetected alterations leading to MSH6 and MSH2 loss cannot be completely excluded. Overall, we interpreted the *MSH6* alteration as likely pathogenic and future studies to validate this by functional assays may be warranted. All of the mutations private to the recurrent tumor were G:C to A:T transition mutations (Additional file 4: Table S2). Other likely pathogenic alterations were identified in the recurrent tumor including *PTEN*, *TP53*, and *KRAS* alterations. The patient was subsequently treated with stereotactic radiosurgery to the occipital tumor site, additional maintenance TMZ cycles, and with combination bevacizumab/irinotecan. The patient died from recurrent disease involving both the temporal and occipital lobes, with overall survival of 26 months.

Case pTMZ-04 from a right frontal lobe tumor in a 12-year-old female aligned to diffuse pediatric-type high-grade glioma, H3-wildytpe and IDH-wildtype by histology and DNA sequencing. Gross total resection was followed by chemoradiotherapy and 18 cycles of maintenance TMZ, with the last documented TMZ given 29 months before recurrence at the anterior margin of the resection cavity. In this case we observed a 3.5-fold increase in TMB from 11.5 mutations/Mb at initial resection to 41 mutations/Mb at recurrence. Interestingly, unlike pTMZ-01 and pTMZ-07 where shared driver mutations could be identified in matched samples, no shared pathogenic driver was identified in pTMZ-04 (Fig. 2 and Additional file 4: Table S2). Also, unlike pTMZ-01 and -07 where acquired MMR gene mutations were identified in TMB-High recurrent tumors, in pTMZ-04 no mutations in the MMR genes were detected in the post-treatment specimen and MMR protein immunohistochemical stains did not show definitive loss. Some heterogeneity in immunoreactivity for PMS2 protein was noted in both the initial and recurrent specimens. However, this did not meet our threshold for protein loss, and NGS on the specimen did not detect *PMS2* mutations. Copy number profiling showed evidence for homozygous deletion of *CDKN2A* and trisomy 18 in both specimens and a few copy number alterations private to the initial or recurrent sample, including copy number gain of *PDGFRA* in the recurrence only (Additional file 6: Fig. S4). Due to the lack of shared driver alterations, discrepant copy number profiles, and *PDGFRA* copy number gain in the recurrence, the possibility of a radiation-associated high-grade astrocytoma was considered. Neither the initial nor the recurrent tumor sample for pTMZ-04 matched to a DNA methylation-based category in the available DKFZ classifiers (versions 11b4 and 12.5).The precise molecular driver of pTMZ-04, the relationship between the initial and recurrent tumor, and the mechanism for increased tumor mutation burden was therefore unresolved in this analysis. The patient died from progressive disease with overall survival of ~ 5 years, approximately 10 months after recurrence.

## Discussion

In this single-institution cohort of pediatric high-grade astrocytomas including 9 pHGG, one pleomorphic xanthoastrocytoma grade 3, and one high-grade astrocytoma NEC, we analyzed pre- and post-TMZ treatment samples by a clinical NGS platform and show evidence for post-treatment increased tumor mutation burden in 3 of 11 cases. We found evidence for post-treatment MMR protein deficiency and MMR gene mutation in two cases, following on this well-established phenomenon in adult patients with IDH-mutant gliomas and IDH-wildtype glioblastoma [15, 16]. Although the number of cases in this study is small, it is rare to have matched tumor samples from patients with pHGG who were treated with TMZ, which makes our cohort relatively unique. Our study takes advantage of a time period when pHGG patients at our institution were treated as part of, or according to, a Children’s Oncology Group clinical trial that included TMZ (NCT00028795) [17]. This was partly based on the success of TMZ regimens in adult high-grade gliomas, and lower toxicity compared to other chemotherapy regimens used at the time. A subsequent clinical trial, NCT00100802, suggested a survival benefit from dual alkylator therapy with lomustine and TMZ in pHGG but also incurred greater toxicity [18]. Current first-line treatment for pHGG is not standardized and depends on many factors including clinical status, extent of resection, tumor type and molecular features, and availability of clinical trials [5]. The decision to treat individual pHGG patients with TMZ, and whether this should be at initial presentation or recurrence, is highly patient- and clinician-dependent.

The implications of post-treatment MMR deficiency and high TMB at recurrence for clinical management in pediatric patients requires further study, and our results should not preclude the use of TMZ in patients when it is clinically indicated. This study supports that retrospective molecular analysis of archived pHGG specimens can resolve histological diagnoses into modern integrated diagnostic categories. This agrees with other recent studies on archived pHGG tumor samples which have used next-generation sequencing, methylation profiling, and copy number analysis to successfully modernize classification of pediatric CNS tumor cohorts [19, 20].

Other studies have analyzed matched tumor samples from primary and recurrent pHGG. Most relevant to our cohort, Salloum et al*.* performed whole-exome sequencing, DNA methylation profiling, and MMR immunohistochemistry analyses on 16 matched primary and recurrent pHGG samples [21]. Similar to the findings in our study, Salloum et al*.* observed that key oncogenic driving alteration (such as H3, IDH, and BRAF mutations) were conserved in primary and recurrent specimens, and that the primary tumor DNA methylation-based subgrouping is maintained at recurrence. A non-statistically-significant trend toward increased TMB at recurrence was observed in the 10 TMZ-treated patients, but no case was described as having acquired post-treatment MMR protein loss or corresponding MMR gene mutation, including one patient with DHG whose tumor had *H3-3A* G34V (p.G35V) mutation. One patient found to have high TMB in both the primary and relapsed/recurrent tumor specimens was found to have a germline *MLH1* splice site mutation. The apparent discrepancy between our study and Salloum et al*.* may be related to the small sample sizes and enrichment of both cohorts for H3 K27-altered DMG which may be less susceptible to treatment-associated MMR deficiency than DHG, as discussed further below.

While the sample size of our cohort is too small for general conclusions, it is interesting that 2 of our 3 post-treatment TMB-High cases are DHG, H3 G34-mutant. In vitro studies have shown that H3 G34 mutations impair H3 lysine 36 methylation, a histone mark that promotes recognition of DNA mismatches by the MutSα dimer composed of MSH2/MSH6 [22, 23]. Haase et al*.* recently reported in vitro and model systems data indicating that H3 G34-mutant high-grade gliomas have impaired DNA mismatch repair at baseline, and delayed activation of DNA damage response pathways in response to ionizing radiation [24]. Interestingly, Haase et al*.* did not observe an increase in mutation rate between H3 G34-mutant and -wildtype tumors, but this would not necessarily include recurrent tumors following chemoradiotherapy and TMZ. These pre-clinical data could suggest that diffuse hemispheric glioma H3 G34-mutant is predisposed to treatment-associated MMR deficiency and increased tumor mutation burden, due to this tumor type having intrinsically compromised baseline DNA mismatch repair.

Diffuse hemispheric glioma H3 G34-mutant is known to have a high rate of *MGMT* promoter hypermethylation compared to other pediatric diffuse gliomas [25, 26]. In a study of adult IDH-mutant diffuse low-grade gliomas, Mathur et al*.* showed that the level of *MGMT* promoter methylation was higher in initial tumors from patients who developed post-treatment recurrences with increased mutation burden, suggesting a potential mechanism for susceptibility to acquired hypermutation in IDH-mutant diffuse gliomas [27]. In our series we identified *MGMT* promoter hypermethylation in six cases, two of which showed increased TMB in the post-treatment recurrence. *MGMT* methylation status was not available for our third TMB-High recurrence [pTMZ-01]. Further studies on *MGMT* promoter methylation in subtypes of pediatric diffuse high-grade gliomas may be warranted to explore the link between the degree of *MGMT* promoter methylation within pHGG tumor types and post-treatment hypermutation.

Our study is limited by the small size of the study cohort, the lack of matched pre- and post-treatment next-generation sequencing data for 2 of our cases, lack of functional characterization of the *MSH6* alteration identified in pTMZ-01, and the unresolved mechanism for the high tumor mutation burden in pTMZ-04. Importantly, our study does not include sequencing of non-tumor tissues to distinguish germline versus somatic alterations. The TMB for pre-treatment samples could be artificially inflated by benign germline or somatic non-tumoral (i.e. passenger) variants. To reduce the impact of this limitation, we focused our analysis and drew conclusions from the *change* in TMB and specific mutations between paired samples. Our study cannot definitively establish if the MMR gene mutations (or other alterations) detected in recurrences are truly absent in the entirety of an initial tumor. Those alterations could be present at very low variant allele frequencies that are below the limit of detection for our assay, or they may have been present in an unsampled portion of the initial tumor.

Several clinical trials have explored TMZ in the treatment of pHGG, as recently summarized in a review by Guerra-Garcia et al. [28]. It is clear from our study and others that trials using histological classifications for enrollment, which would have been the diagnostic standard at the time, will actually be a mixed cohort of tumors with distinct molecular drivers, natural histories, and potentially different susceptibilities to various treatments. Detailed histological, genetic, and epigenetic re-appraisal of trial results where archived tissue is available can be highly informative and could inform subtype-specific treatment responses and/or resistance mechanisms [26]. Results from our cohort suggest that assessment of post-treatment TMB and MMR deficiency should be carefully scrutinized in pHGG, particularly in DHG H3 G34-mutant tumors.

### Supplementary Information

**Additional file 1**. **Figure S1**. Radiologic features of initial tumor presentation and post-treatment recurrences.

**Additional file 2**. Results of genome-wide DNA methylation-based profiling.

**Additional file 3**.** Figure S2**. Histologic features of pTMZ-09, high-grade astrocytoma NEC. Scale bars are 100 microns (**A**–**C**) and 50 microns (**D**).

**Additional file 4**. Alterations identified by next-generation DNA sequencing.

**Additional file 5**.** Figure S3**. Structural view of the MutSα heterodimer highlighting proline 531, and the location of a predicted MSH2/MSH6 hydrogen bond (black arrow on inset).

**Additional file 6**.** Figure S4**. Copy number plots from DNA methylation arrays for pre- and post-TMZ case 4.

## Data Availability

The datasets supporting the conclusions of this article are included within the article and its additional files. Raw data files are available upon reasonable request from the corresponding author.

## Abbreviations

CNS: Central nervous system
DHG: Diffuse hemispheric glioma, H3 G34-mutant
DMG: Diffuse midline glioma, H3 K27-altered
DNA-MP: DNA methylation-based profiling
IDH: Isocitrate dehydrogenase
MMR: Mismatch repair
NEC: Not elsewhere classified
NGS: Next-generation sequencing
OHSU: Oregon Health & Science University
pHGG: Pediatric-type diffuse high-grade glioma
TMB: Tumor mutation burden
TMZ: Temozolomide
WHO: World Health Organization
WHO CNS 5: World Health Organization classification of central nervous system tumors, 5th edition (2021)
