# Supplementary figures and images for: Molecular profiling of pre- and post-treatment pediatric high-grade astrocytomas reveals acquired increased tumor mutation burden in a subset of recurrences
Source: Acta Neuropathol Commun. 2023 Sep 5;11:143. doi: 10.1186/s40478-023-01644-4 (PMC10481558; doi:10.1186/s40478-023-01644-4)

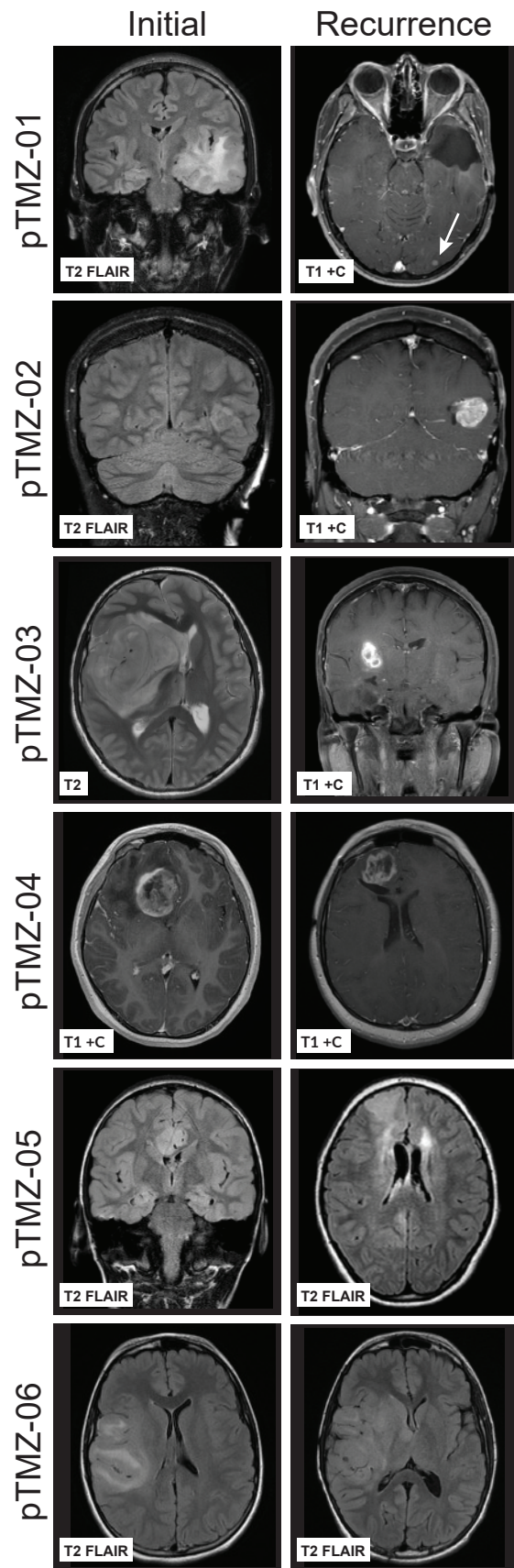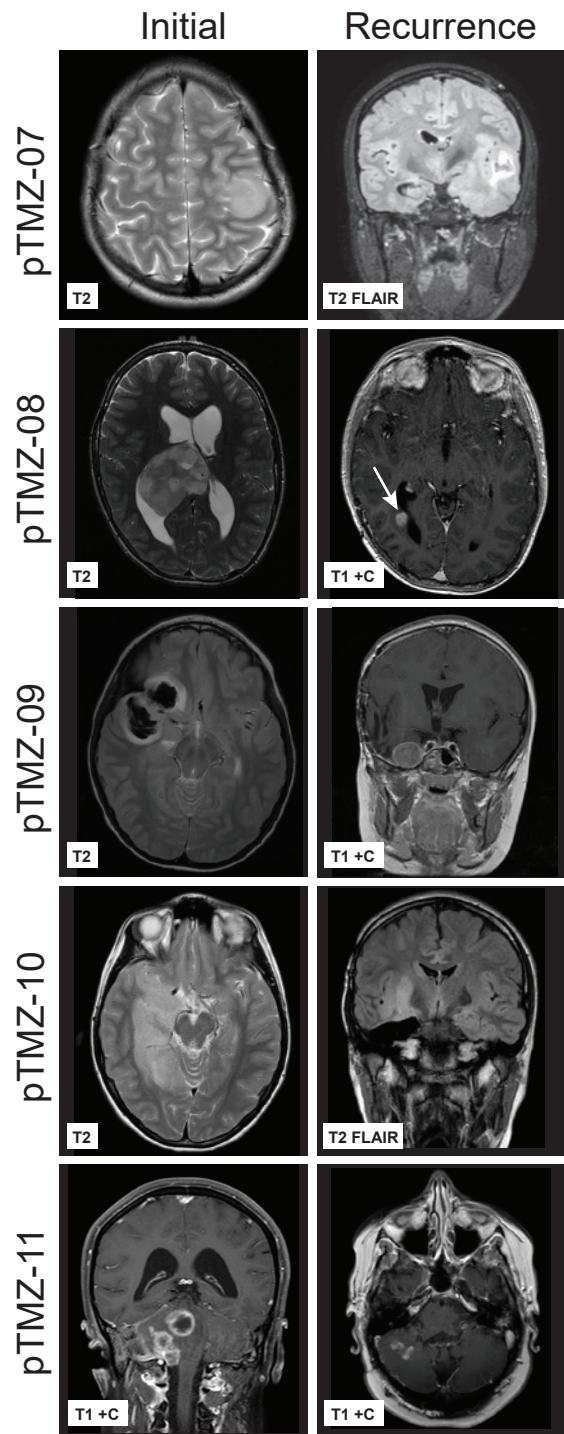

Supplement: Supplementary file 1 — Additional file 1. Figure S1. Radiologic features of initial tumor presentation and post-treatment recurrences. [file 40478_2023_1644_MOESM1_ESM.pdf]

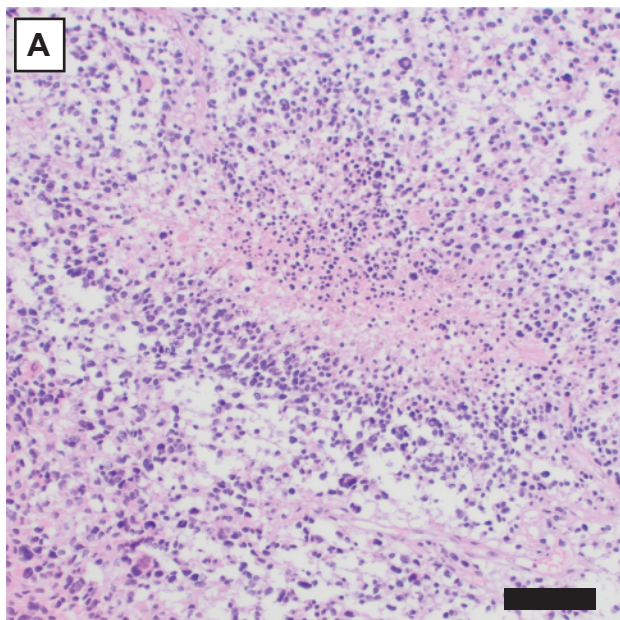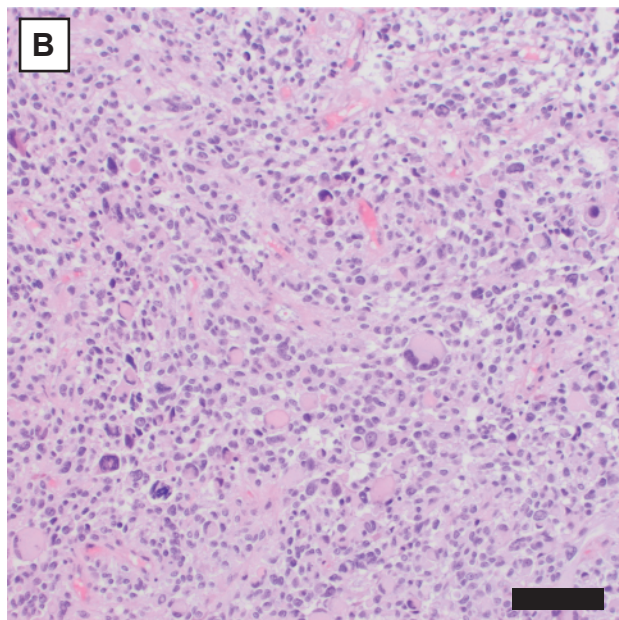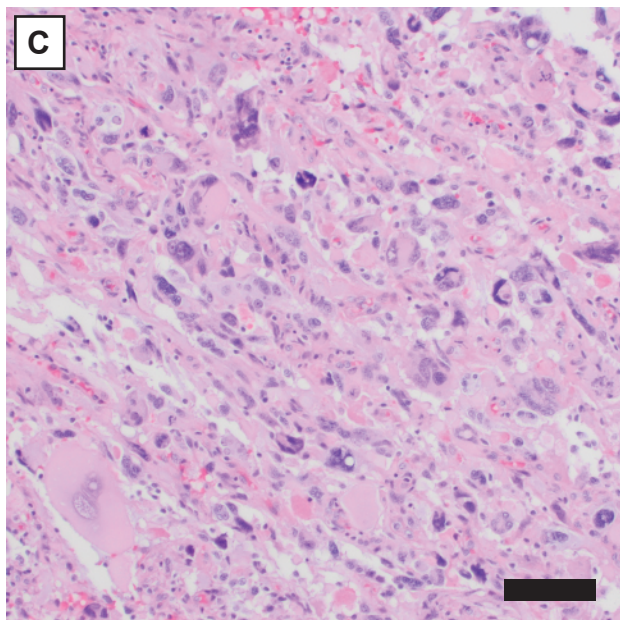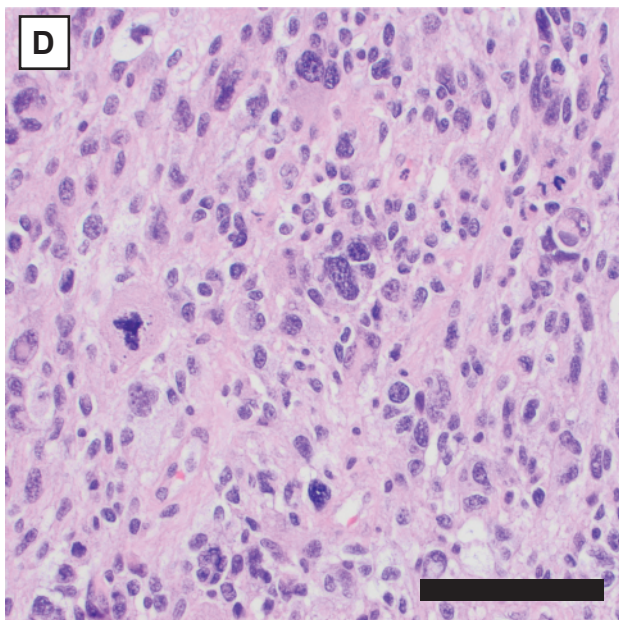

Supplement: Supplementary file 3 — Additional file 3. Figure S2. Histologic features of pTMZ-09, high-grade astrocytoma NEC. Scale bars are 100 microns (A–C) and 50 microns (D). [file 40478_2023_1644_MOESM3_ESM.pdf]

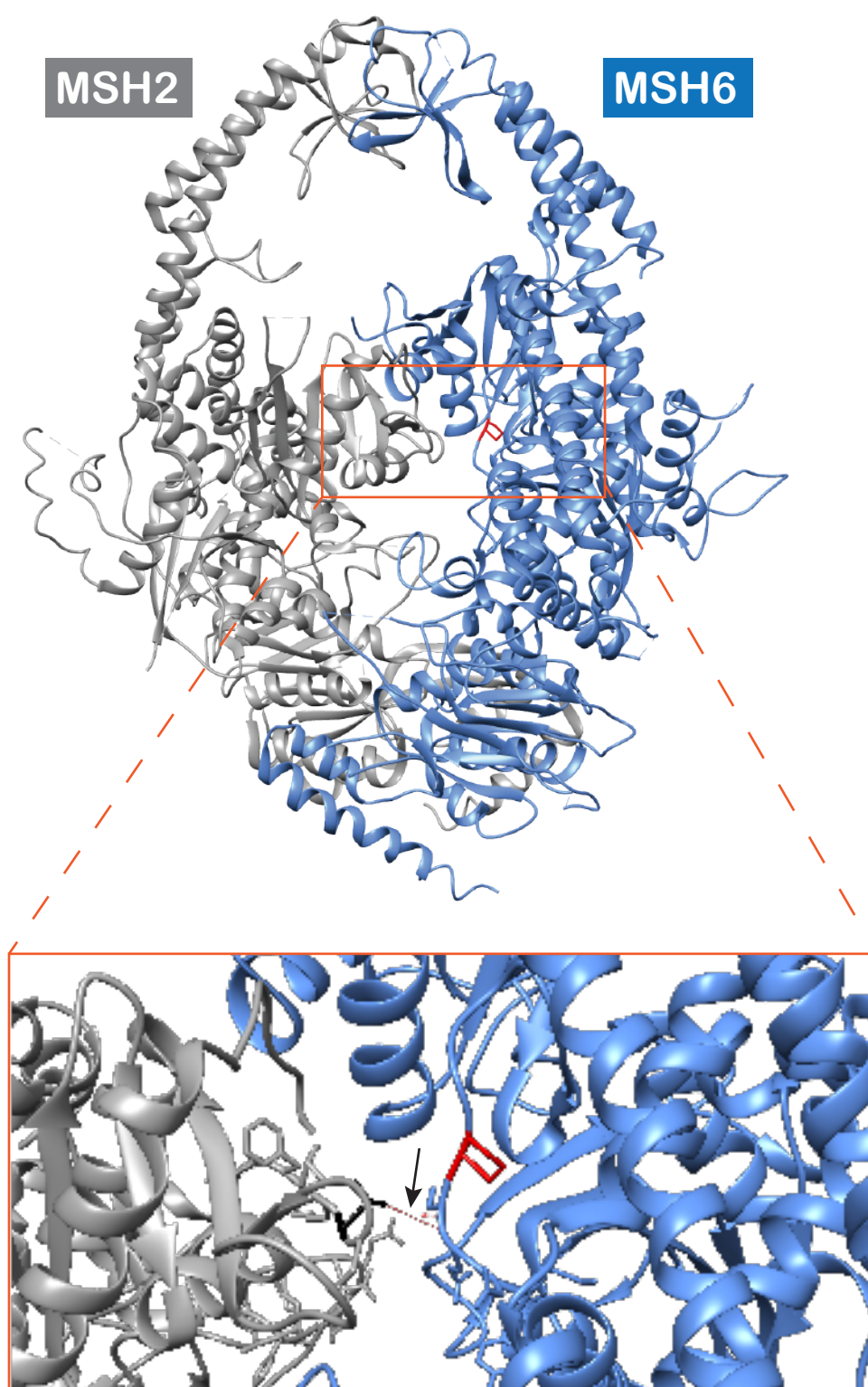

Supplement: Supplementary file 5 — Additional file 5. Figure S3. Structural view of the MutSα heterodimer highlighting proline 531, and the location of a predicted MSH2/MSH6 hydrogen bond (black arrow on inset). [file 40478_2023_1644_MOESM5_ESM.pdf]

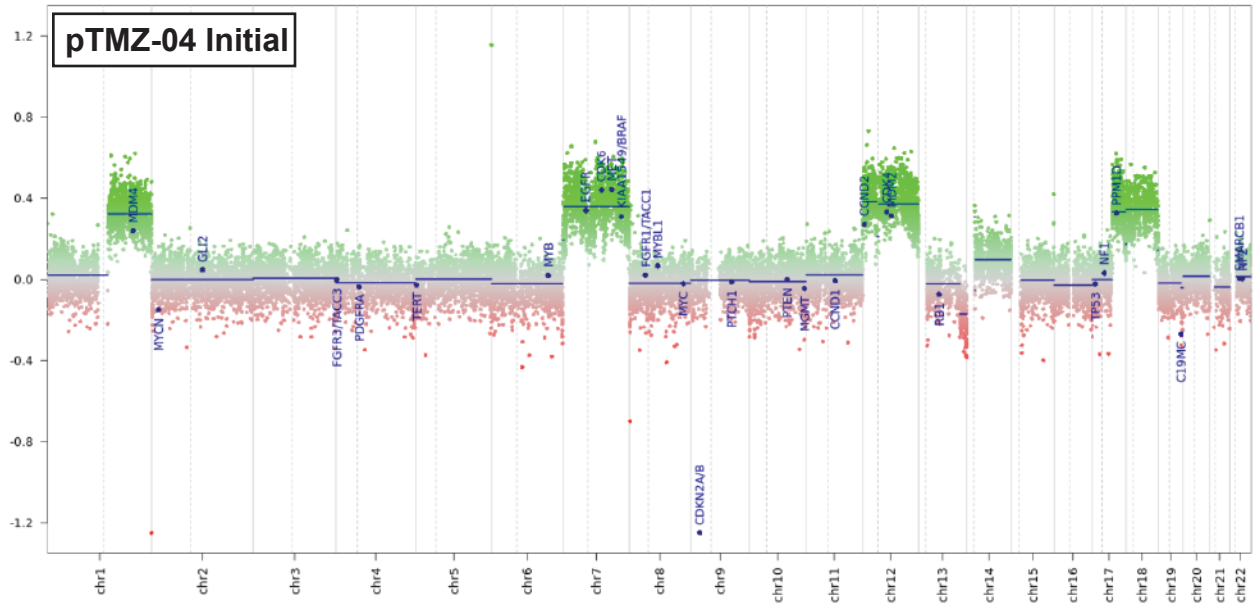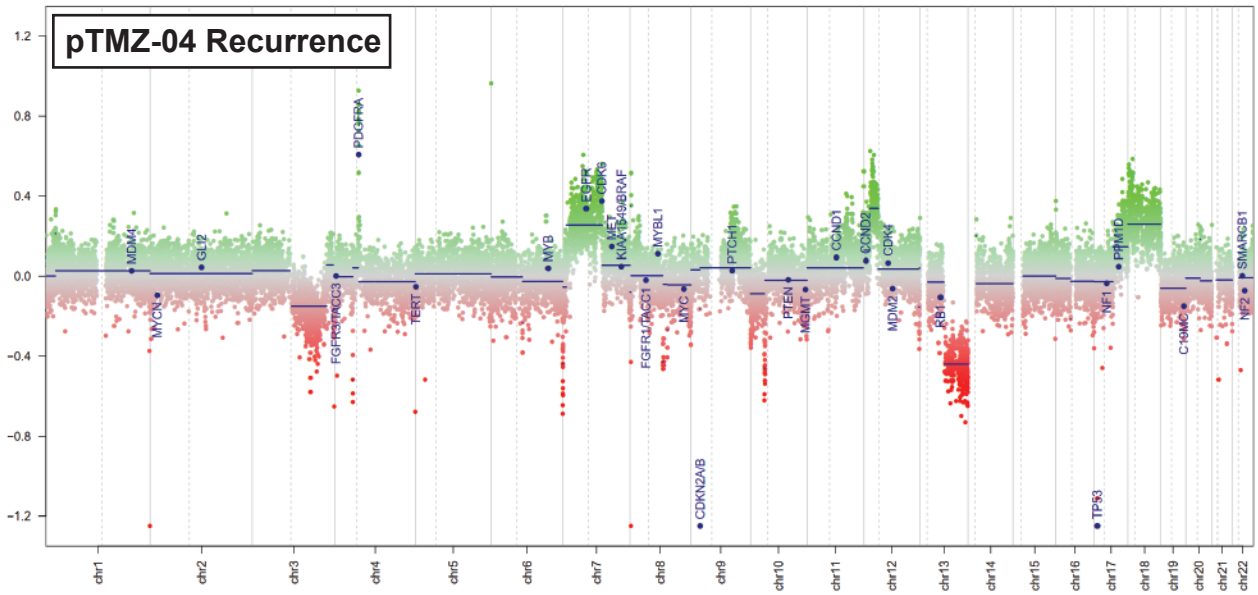

Supplement: Supplementary file 6 — Additional file 6. Figure S4. Copy number plots from DNA methylation arrays for pre- and post-TMZ case 4. [file 40478_2023_1644_MOESM6_ESM.pdf]
