# Supplementary material for: Molecular profiling of pre- and post-treatment pediatric high-grade astrocytomas reveals acquired increased tumor mutation burden in a subset of recurrences
Source: Acta Neuropathol Commun. 2023 Sep 5;11:143. doi: 10.1186/s40478-023-01644-4 (PMC10481558; doi:10.1186/s40478-023-01644-4)
Supplement: Supplementary file 2 — Additional file 2. Results of genome-wide DNA methylation-based profiling. [file 40478_2023_1644_MOESM2_ESM.docx]

| **Case** | **Treatment** | **Result** | **Highest Class** | **Score** | **MGMT status** | **Interpretation** |
| --- | --- | --- | --- | --- | --- | --- |
| pTMZ-01 | N/A | N/A | N/A | N/A | N/A | Technical failure on both pre- and post-TMZ samples |
|  |  |  |  |  |  |  |
| pTMZ-02 | Post | Match | PXA | 0.99 | Unmethylated | Concordant to histology and genetics, *CDKN2A* deletion |
|  |  |  |  |  |  |  |
| pTMZ-03 | Pre | Match | pedHGG_MYCN | 1.00 | Unmethylated | Concordant to histology and genetics, *MYCN* amplified |
| pTMZ-03 | Post | Match | pedHGG_MYCN | 1.00 | Unmethylated | Concordant to histology and genetics, *MYCN* amplified |
|  |  |  |  |  |  |  |
| pTMZ-04 | Pre | No match | GBM_RTK2 | 0.30 | Methylated | No matching methylation class, trisomy 7, *CDKN2A* deletion |
| pTMZ-04 | Post | No match | GBM_RTK1 | 0.35 | Methylated | No matching methylation class, *PDGFRA* copy number gain, *CDKN2A* deletion |
|  |  |  |  |  |  |  |
| pTMZ-05 | Pre | No match | DMG_EGFR | 0.23 | Unmethylated | Possible low sample quality or technical limitation |
| pTMZ-05 | Post | Match | DMG_EGFR | 1.00 | Unmethylated | Concordant to histology and genetics |
|  |  |  |  |  |  |  |
| pTMZ-06 | Post | Match | pedHGG_RTK2A | 1.00 | Unmethylated | Concordant to histology and genetics |
|  |  |  |  |  |  |  |
| pTMZ-07 | Pre | Match | DHG_G34 | 1.00 | Methylated | Concordant to histology and genetics |
| pTMZ-07 | Post | Match | DHG_G34 | 1.00 | Methylated | Concordant to histology and genetics |
|  |  |  |  |  |  |  |
| pTMZ-08 | Pre | No match | MB_SHH_2 | 0.37 | Methylated | Possible low sample quality or technical limitation |
| pTMZ-08 | Post | No match | MB_SHH_2 | 0.48 | Methylated | Possible low sample quality or technical limitation |
|  |  |  |  |  |  |  |
| pTMZ-09 | Pre | No match | IHG | 0.27 | Methylated | Possible low sample quality or technical limitation |
| pTMZ-09 | Post | No match | pedHGG_A | 0.19 | Methylated | Possible low sample quality or technical limitation |
|  |  |  |  |  |  |  |
| pTMZ-10 | Pre | Match | DMG_EGFR | 1.00 | Methylated | Concordant to histology and genetics |
| pTMZ-10 | Post | Match | DMG_EGFR | 0.99 | Methylated | Concordant to histology and genetics |
|  |  |  |  |  |  |  |
| pTMZ-11 | Pre | Match | A_IDH_HG | 1.00 | Methylated | Concordant to histology and genetics |
| pTMZ-11 | Post | No match | A_IDH_HG | 0.86 | Methylated | Near-match, concordant to histology and genetics |
